# Associations between Clinical Findings and Severity of Diffuse Idiopathic Skeletal Hyperostosis in Patients with Ossification of the Posterior Longitudinal Ligament

**DOI:** 10.3390/jcm10184137

**Published:** 2021-09-14

**Authors:** Takashi Hirai, Soraya Nishimura, Toshitaka Yoshii, Narihito Nagoshi, Jun Hashimoto, Kanji Mori, Satoshi Maki, Keiichi Katsumi, Kazuhiro Takeuchi, Shuta Ushio, Takeo Furuya, Kei Watanabe, Norihiro Nishida, Kota Watanabe, Takashi Kaito, Satoshi Kato, Katsuya Nagashima, Masao Koda, Hiroaki Nakashima, Shiro Imagama, Kazuma Murata, Yuji Matsuoka, Kanichiro Wada, Atsushi Kimura, Tetsuro Ohba, Hiroyuki Katoh, Masahiko Watanabe, Yukihiro Matsuyama, Hiroshi Ozawa, Hirotaka Haro, Katsushi Takeshita, Morio Matsumoto, Masaya Nakamura, Masashi Yamazaki, Yu Matsukura, Hiroyuki Inose, Atsushi Okawa, Yoshiharu Kawaguchi

**Affiliations:** 1 Department of Orthopaedic Surgery, Tokyo Medical and Dental University, Bunkyo-ku, Tokyo 113-8510, Japan; yoshii.orth@tmd.ac.jp (T.Y.); 0123456789jun@gmail.com (J.H.); ushiorth20@gmail.com (S.U.); matsukura.orth@tmd.ac.jp (Y.M.); inose.orth@tmd.ac.jp (H.I.); okawa.orth@tmd.ac.jp (A.O.); 2 Department of Orthopedic Surgery, School of Medicine, Keio University, Shinjuku, Tokyo 160-8582, Japan; soraya.nishimura@gmail.com (S.N.); nagoshi@2002.jukuin.keio.ac.jp (N.N.); kw197251@keio.jp (K.W.); morio@a5.keio.jp (M.M.); masa@a8.keio.jp (M.N.); 3 Department of Orthopaedic Surgery, Shiga University of Medical Science, Ōtsu 520-2192, Japan; kanchi@belle.shiga-med.ac.jp; 4 Department of Orthopedic Surgery, School of Medicine, Chiba University Graduate, Chiba 260-0856, Japan; satoshi.maki@chiba-u.jp (S.M.); takeo251274@yahoo.co.jp (T.F.); 5 Department of Orthopedic Surgery, Niigata University Medical and Dental General Hospital, Niigata 951-8520, Japan; kkatsu_os@yahoo.co.jp (K.K.); keiwatanabe_39jp@live.jp (K.W.); 6 National Hospital Organization Okayama Medical Center, Department of Orthopedic Surgery, Okayama 701-1192, Japan; takeuchi@okayamamc.jp; 7 Department of Orthopedic Surgery, Graduate School of Medicine, Yamaguchi University, Yamaguchi 755-8505, Japan; nishida3@yamaguchi-u.ac.jp; 8 Department of Orthopaedic Surgery, Graduate School of Medicine, Osaka University, Suita 565-0871, Osaka, Japan; takashikaito@gmail.com; 9 Department of Orthopedic Surgery, Graduate School of Medical Sciences, Kanazawa University, Kanazawa 920-1192, Japan; skato323@gmail.com; 10 Department of Orthopedic Surgery, Faculty of Medicine, University of Tsukuba, Tsukuba 305-8577, Japan; katsu_n103@yahoo.co.jp (K.N.); masaokod@gmail.com (M.K.); masashiy@md.tsukuba.ac.jp (M.Y.); 11 Department of Orthopedics, Graduate School of Medicine, Nagoya University, 65 Tsurumai, Shouwa-ku, Nagoya 466-8560, Japan; hirospine@med.nagoya-u.ac.jp (H.N.); imagama@med.nagoya-u.ac.jp (S.I.); 12 Department of Orthopedic Surgery, Tokyo Medical University, Shinjuku, Tokyo 160-8402, Japan; kaz.mur26@gmail.com (K.M.); yuji_kazu77@yahoo.co.jp (Y.M.); 13 Department of Orthopaedic Surgery, Graduate School of Medicine, Hirosaki University, Hirosaki 036-8562, Japan; wadak39@hirosaki-u.ac.jp; 14 Department of Orthopedics, Jichi Medical University, Shimotsuke 329-0498, Japan; akimura@jichi.ac.jp (A.K.); dtstake@gmail.com (K.T.); 15 Department of Orthopedic Surgery, University of Yamanashi, Chuo 409-3898, Japan; tooba@yamanashi.ac.jp (T.O.); haro@yamanashi.ac.jp (H.H.); 16 Department of Orthopedic Surgery, Surgical Science, School of Medicine, Tokai University, Isehara 259-1193, Japan; hero@tokai-u.jp (H.K.); masahiko@is.icc.u-tokai.ac.jp (M.W.); 17 Department of Orthopedic Surgery, School of Medicine, Hamamatsu University, Hamamatsu 431-3125, Japan; spine-yu@hama-med.ac.jp; 18 Department of Orthopaedic Surgery, Tohoku Medical and Pharmaceutical University, Sendai 983-8536, Japan; hozawa@med.tohoku.ac.jp; 19 Department of Orthopedic Surgery, Faculty of Medicine, University of Toyama, Toyama 930-0194, Japan; zenji@med.u-toyama.ac.jp

**Keywords:** cervical spine, clinical findings, computed tomography, diffuse idiopathic skeletal hyperostosis, ossification of the posterior longitudinal ligament, pain, patient-reported outcomes, whole spine

## Abstract

Background: This study investigated how diffuse idiopathic skeletal hyperostosis (DISH) influences clinical characteristics in patients with cervical ossification of the posterior longitudinal ligament (OPLL). Although DISH is considered unlikely to promote neurologic dysfunction, this relationship remains unclear. Methods: Patient data were prospectively collected from 16 Japanese institutions. In total, 239 patients with cervical OPLL were enrolled who had whole-spine computed tomography images available. The primary outcomes were visual analog scale pain scores and the results of other self-reported clinical questionnaires. Correlations were sought between clinical symptoms and DISH using the following grading system: 1, DISH at T3-T10; 2, DISH at both T3–10 and C6–T2 and/or T11–L2; and 3, DISH beyond the C5 and/or L3 levels. Results: DISH was absent in 132 cases, grade 1 in 23, grade 2 in 65, and grade 3 in 19. There were no significant correlations between DISH grade and clinical scores. However, there was a significant difference in the prevalence of neck pain (but not in back pain or low back pain) among the three grades. Interestingly, DISH localized in the thoracic spine (grade 1) may create overload at the cervical spine and lead to neck pain in patients with cervical OPLL. Conclusion: This study is the first prospective multicenter cross-sectional comparison of subjective outcomes in patients with cervical OPLL according to the presence or absence of DISH. The severity of DISH was partially associated with the prevalence of neck pain.

## 1. Introduction

Ossification of the spinal ligaments impairs spinal mobility and occasionally leads to a spinal disorder [1,2]. Ossification of the posterior longitudinal ligament (OPLL) is common in Asian countries and can cause severe myelopathy [3]. Diffuse idiopathic skeletal hyperostosis (DISH), which is defined as ossification of the anterior longitudinal ligament bridging at least four vertebral segments of the thoracolumbar spine [4,5], has also been recognized as a pathological feature in patients predisposed to ossification and often coincides with the presence of OPLL [6,7,8,9,10]. Although DISH has been widely regarded as an asymptomatic disorder, it is unclear how it affects symptoms related to the whole spine. Few studies have compared patients with and without DISH in terms of clinical symptoms. Therefore, the Japanese Multicenter Research Organization for Ossification of the Spinal Ligament (JOSL), established a nationwide patient registry to prospectively collect the clinical and radiologic data, including whole-spine computed tomography (CT) scans, of OPLL patients. Using data from this registry, this paper focuses on differences in clinical and radiological findings between patients with and without DISH. We further sought to identify any significant associations between clinical symptoms and the severity of DISH in these patients based on patient-reported outcomes.

## 2. Materials and Methods

### 2.1. Patients and Methods

This multicenter prospective cross-sectional study used data from 16 member institutions of the JOSL established by the Japan Ministry of Health, Labour, and Welfare. The inclusion criteria were as follows: age ≥20 years; diagnosis of cervical OPLL based on radiographic findings; symptoms such as neck pain and upper and/or lower extremity numbness regardless of whether surgery was required, clumsiness, and gait disturbance; a visit made to a participating institution for symptoms between September 2015 and December 2017; and whole-spine CT scans available to determine the location of ossified lesions in the spine. The only exclusion criterion was a history of cervical spine surgery for OPLL. The study was approved by the institutional review board of each participating institution and conducted in accordance with the relevant guidelines and regulations.

### 2.2. Clinical Evaluation

Basic demographic and clinical data of patients were collected, including age, sex, diabetes mellitus (DM) status, body mass index (BMI), and presence of neck pain, back pain, and/or low back pain (LBP). Clinical status was evaluated using the following measures: cervical Japanese Orthopaedic Association (JOA) score [11], which is used for functional assessment of patients with cervical myelopathy, JOA Cervical Myelopathy Evaluation Questionnaire (JOA-CMEQ) [12], which assesses the function of the cervical spine, upper and lower extremities, and bladder as well as quality of life; and the JOA Back Pain Evaluation Questionnaire (JOA-BPEQ) [13], which assesses lumbar spine function, social dysfunction, mentality, locomotive function, and body pain. The degree of pain or stiffness in the neck or shoulders, pain or numbness in the arms or hands, and LBP was evaluated using a visual analog scale (VAS).

### 2.3. Radiologic Evaluations

**Figure 1:**
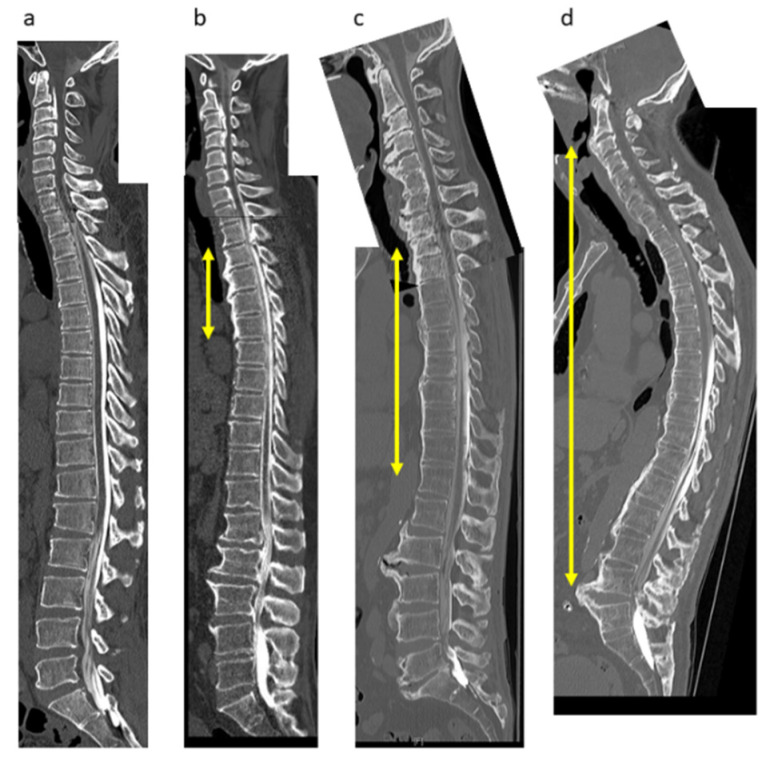
DISH grading system. (**a**) No DISH; (**b**) Grade 1 (bony bridge at T3–T6); (**c**) Grade 2 (T2–T12); (**d**) Grade 3 (C2–L5). DISH, diffuse idiopathic skeletal hyperostosis.

CT images of the whole spine were collected for each patient. The images included the cervical, thoracic, and lumbosacral segments, spanning the occipital bone to the sacrum. The incidence of OPLL in the cervical spine from the clivus to C7 and in other spinal regions from T1 to S1 was evaluated on mid-sagittal CT images. Blinded to clinical outcomes, six senior spine surgeons (S.U., K.M., S.M., K.K., N.N., and K.T.) independently evaluated the images, as described previously [13]. OPLL was assessed as DISH if it completely bridged at least four contiguous adjacent vertebral bodies anywhere in the spine based on the criteria established by Resnick and Niwayama [5]. In accordance with a previous report [10], DISH was classified as follows: grade 1, DISH at T3–T10; grade 2, DISH at both T3–10 and C6–T2 and/or T11–L2; and grade 3, DISH extends beyond the C5 and/or L3 levels (Figure 1). To identify any significant differences in clinical findings, we compared patients with and without DISH and those with DISH according to grade. In addition, the ossification of the posterior longitudinal ligament index (OP-index), defined as the number of levels with OPLL in the whole spine [6], was also calculated for each patient.

## 3. Results

### 3.1. Demographic and Clinical Data 

The demographic and clinical characteristics of the patients are shown according to DISH status in Table 1. There was no significant difference in age, BMI, DM status, or cervical JOA score between the group with DISH (*n* = 107) and the group without DISH (*n* = 132). Table 1 shows the prevalence of pain and the JOA-CMEQ, JOA-BPEQ, and VAS scores for each domain. There was no significant between-group difference in these patient-reported outcomes except for lumbar spine function; however, there was a significant difference in the OP-index value.

### 3.2. Demographic and Clinical Characteristics by DISH Grade

**Figure 2:**
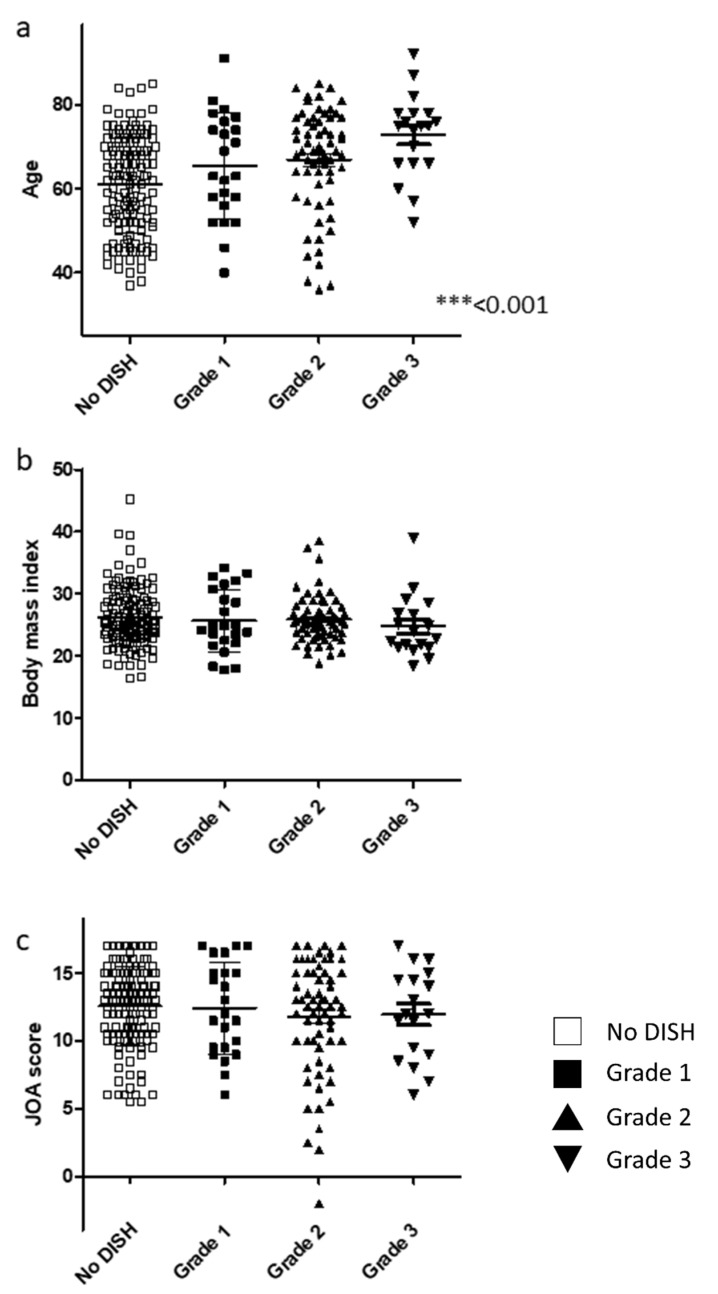
Relationship between basic demographic and clinical findings and DISH grade. (**a**) Patient age. (**b**) Body mass index. (**c**) JOA score. DISH, diffuse idiopathic skeletal hyperostosis; JOA, Japanese Orthopaedic Association.

Patient demographics are shown according to DISH grade in Table 2 and Figure 2. There was a significant between-group difference in age (Figure 2a) but not in the sex distribution. No significant between-group difference was found in BMI (Figure 2b), DM status, or cervical JOA score among the three grades (Figure 2c). There was a significant correlation between the OP-index and DISH grade (Table 2).

### 3.3. Severity of DISH Was Not Associated with Myelopathic Symptoms or Lumbar Spine Function in Patients with Cervical OPLL

**Figure 3:**
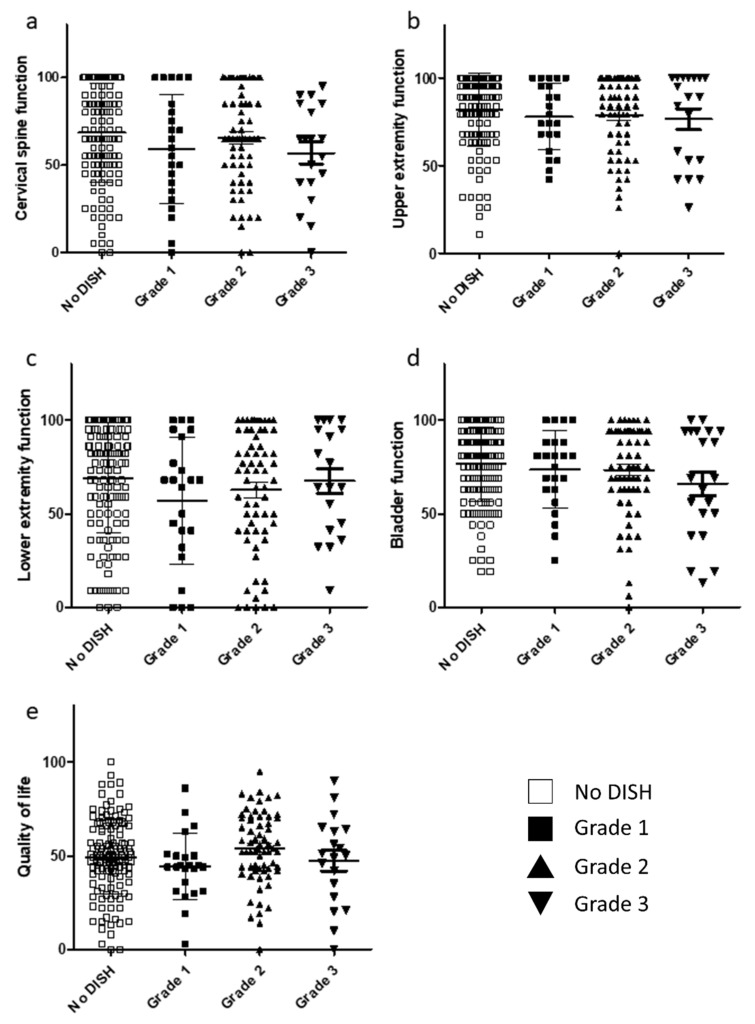
Relationship between JOA-CMEQ scores and DISH grade. (**a**) Cervical function. (**b**) Upper extremity function. (**c**) Lower extremity function. (**d**) Bladder function. (**e**) Quality of life. DISH, diffuse idiopathic skeletal hyperostosis; JOA-CMEQ, Japanese Orthopaedic Association Cervical Myelopathy Evaluation Questionnaire.

**Figure 4:**
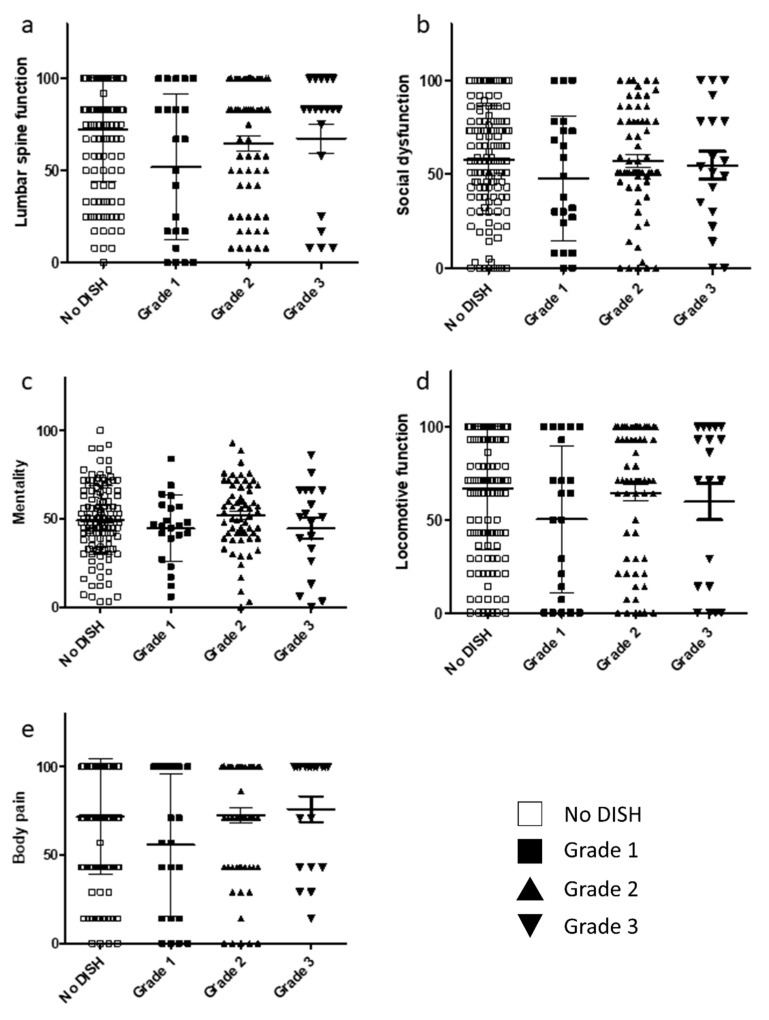
Relationship between JOA-BPEQ and DISH grade. (**a**) Lumbar function. (**b**) Social dysfunction. (**c**) Mentality. (**d**) Locomotive function. (**e**) Body pain. DISH, diffuse idiopathic skeletal hyperostosis; JOA-BPEQ, Japanese Orthopaedic Association Back Pain Evaluation Questionnaire.

The score for each item in the JOA-CMEQ and JOA-BPEQ was evaluated to assess whether the severity of DISH in terms of cervical myelopathy and lumbar spine function affects the ability to perform activities of daily living. There were no significant correlations among the four groups for JOA-CMEQ scores (Figure 3a–e). Similarly, there were no significant differences among the three DISH grades in terms of lumbar spine function, social dysfunction, mentality, locomotive function, and body pain (Figure 4a–e).

### 3.4. Degree of DISH Correlated Negatively with Prevalence of Neck Pain but Not Back Pain or LBP in Patients with Cervical OPLL

The prevalence of neck pain was significantly correlated with degree of DISH, but back pain and LBP were not (Table 3). Furthermore, although there was no statistically significant difference in LBP among the three grades of DISH, LBP tended to decrease with increasing grade.

**Figure 5:**
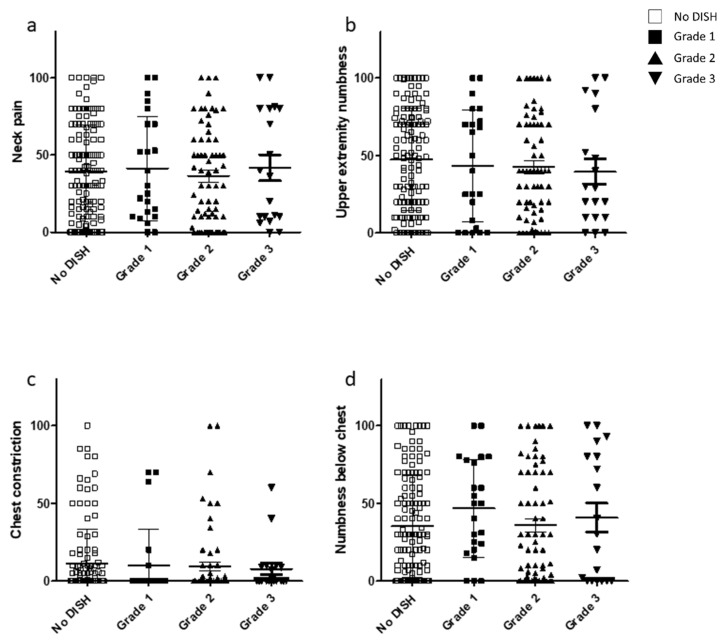
Relationship between the VAS scores included in the JOA-CMEQ and DISH grade. VAS scores for (**a**) neck pain, (**b**) upper extremity numbness, (**c**) chest constriction, and (**d**) numbness below the chest. DISH, diffuse idiopathic skeletal hyperostosis; JOA-CMEQ, Japanese Orthopaedic Association Cervical Myelopathy Evaluation Questionnaire; VAS, visual analog scale.

**Figure 6:**
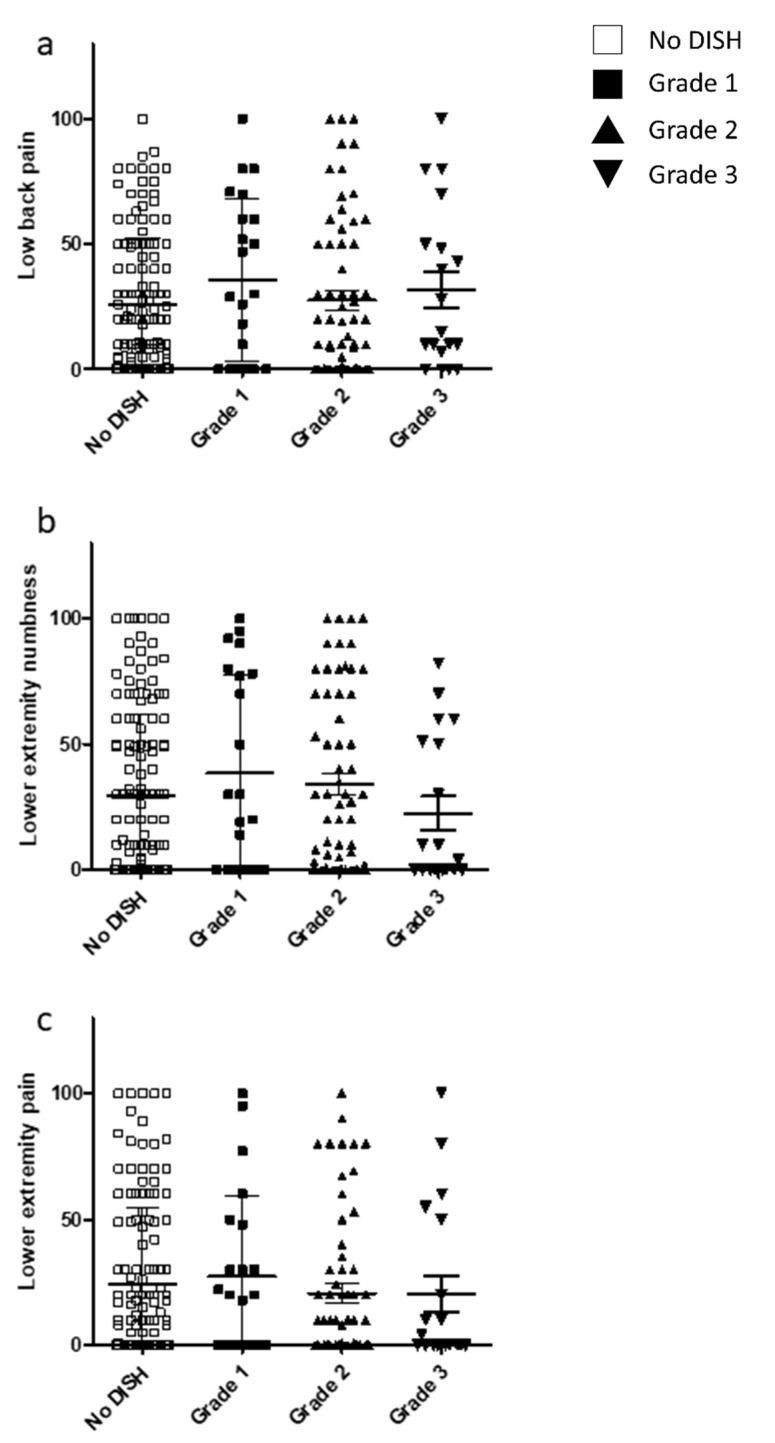
Relationship between the VAS scores included in the JOA-BPEQ and DISH grade. VAS scores for (**a**) low back pain, (**b**) lower extremity numbness, and (**c**) lower extremity pain. DISH, diffuse idiopathic skeletal hyperostosis; JOA-BPEQ, Japanese Orthopaedic Association Back Pain Evaluation Questionnaire; VAS, visual analog scale.

VAS scores from the JOA-CMEQ and JOA-BPEQ were investigated to clarify the relationship between degree of DISH and pain associated with cervical myelopathy. However, no significant difference was found in the VAS scores among the three DISH grades (Figure 5 and Figure 6).

## 4. Discussion

DISH is a systemic condition characterized by ossification of ligaments and entheses throughout the body. Considered to be mostly an asymptomatic condition, DISH was largely ignored by clinicians and researchers until the 1990s. However, it is now known that DISH can sometimes result in specific symptoms, including back pain [14], stiffness [15], reduced range of articular motion [4] and dysphagia [16]. Notably, energy cannot be distributed over multiple segments in patients with DISH. Therefore, even minor trauma can lead to an unstable spinal fracture. A retrospective study [17,18] reviewed 289 patients with DISH-related spinal fractures and demonstrated that these fractures frequently resulted in spinal cord injury and were sometimes associated with mortality. That study also found that the diagnosis was often delayed, leading to unexpected impairment of neurologic status, especially in patients with a thoracolumbar fracture. Therefore, it is important to recognize the presence of this pathology and the associated risks even after minor trauma, given that DISH creates longer bony lever arms, which increase spinal instability at the fracture site when a fracture occurs. 

Patients with cervical OPLL often have ossification of other spinal ligaments, including the ligamentum flavum, anterior longitudinal ligament, and the interspinous and supraspinous ligaments. In earlier studies [4,19,20,21,22], 25–50% of patients with cervical OPLL had DISH. A previous retrospective study by our group [10] revealed that DISH was distributed primarily in the middle thoracic spine in younger patients but could extend to the cervical and/or lumbar spine in older patients. Toyoda et al. [23] reported that the prevalence of DISH increased with age in whole-spine radiographs of 345 patients in whom spinal surgery was required. Older patients in the present study also had a more severe DISH grade. Although a further longitudinal study is needed, the evidence to date suggests that ossification of the anterior longitudinal ligament might progress gradually from the thoracic spine to the cervical spine and lumbar spine with aging.

DISH has been recognized to be not only a structural abnormality in the human thoracic spine but also a result of metabolic syndrome. Okada et al. [24] compared subjects with and without DISH and demonstrated that the prevalence of metabolic syndrome was significantly higher in patients with DISH than in those without DISH (28.9% vs. 16.0%). Furthermore, using abdominal CT, Lantsman et al. [25] showed that areas of visceral fat were larger in patients with DISH than in healthy controls. Although there were no significant associations in terms of the prevalence of DM between patients with and without DISH or among the three grades in the present study, the onset and extent of DISH may be associated with a systemic metabolic disorder.

This prospective multicenter study is the first to investigate subjective outcomes in patients with cervical OPLL according to DISH status. Although we collected patient-reported outcomes for activities of daily living, we found no DISH-related differences in patients with cervical OPLL. These findings are consistent with the opinion of some clinicians that DISH should be considered a state rather than a disease [26]. DISH may be present not only by itself but can also accompany ossification of other spinal ligaments that often lead to spinal cord disorders [13,27]. Therefore, in the present study, to reduce selection bias in this regard, we enrolled only patients with cervical OPLL. Therefore, we believe that DISH does not directly impair neurologic status or quality of life.

Several studies have investigated the association between presence of DISH and physical pain. Mata et al. [28] compared clinical symptoms in 56 patients with DISH, 43 control patients with lumbar spondylosis, and healthy volunteers and demonstrated that patients with DISH were more likely to report a history of upper extremity pain, medial epicondylitis of the elbow, enthesitis of the patella or heel, and dysphagia than were patients with lumbar spondylosis. They also reported that neck rotation and thoracic movements were more limited in the patients with DISH than in the patients with spondylosis or the healthy controls, and lumbar movement was more restricted in the patients with DISH than in the healthy controls. However, the findings of a similar study were contradictory. Schlapbach et al. [29] demonstrated that the radiological findings for DISH were not associated with an increased frequency of back pain and had no clinical relevance. Moreover, Holton et al. [30] randomly collected data for 298 elderly men from a surveillance cohort of 5995 men and demonstrated that the frequency of LBP was reduced in 126 men with DISH compared with 172 men without DISH based on North American Spine Society questionnaires for back and neck pain. We have also shown that patients with continuous OPLL are less likely to have neck pain than those with other types of ossification in which the cervical spine has more mobility than in continuous OPLL [27]. Similarly, the present study revealed that the prevalence of neck pain decreased with increasing DISH grade. Given that patients with DISH are often found to have ossification of other spinal ligaments, the structural change caused by DISH alone cannot always explain their clinical status. Indeed, in this study, there was a significant increase in the OP-index value with increasing DISH grade, which may be a confounding factor. However, the present findings suggest that segmental motion at unstable intervertebral levels rather than bony bridging segments is likely to cause pain and that neck pain is likely to be less severe in patients with a more severely ankylosed spine (DISH grade 3) than in those with a less restricted spine. Therefore, DISH localized in the thoracic spine (grade 1) may create overload at the cervical and lumbar spine and lead to neck pain and LBP.

This study has several limitations. First, it was a cross-sectional cohort study of a specific disease and not population-based. Second, the study was not longitudinal and thus cannot reach conclusions on causality. Third, the presence of DISH was evaluated only on reconstructed sagittal CT images with no review of bony bridges at the lateral portion of the intervertebral segments. Fourth, we could not determine whether mobility of the segment adjacent to DISH affects neck pain or LBP. Fifth, the JOA-CMEQ could not evaluate pain states in detail. Further studies are required in the general population to clarify these clinical questions and eliminate confounding factors in terms of each spinal ligament. However, despite these limitations, we believe that our findings provide important information on the clinical features of DISH in patients with cervical OPLL.

## 5. Conclusions

This study is the first prospective multicenter cross-sectional comparison of subjective outcomes in patients with cervical OPLL according to the presence or absence of DISH. There were no significant correlations between DISH grade and clinical scores. However, there was a significant difference in the prevalence of neck pain among the three grades, albeit not in the prevalence of back pain or LBP. Interestingly, DISH localized in the thoracic spine (grade 1) could create overload at the cervical spine and lead to neck pain in patients with cervical OPLL.

## Figures and Tables

**Table 1:** Demographic and clinical data for patients with OPLL according to presence or absence of DISH.

	No DISH (*n* = 132)	DISH (*n* = 107)	*p*-Value
Age (years)	60.9 ± 11.6	67.6 ± 12.1	<0.001 ***
Male (%)	61.4	76.6	0.01 *
Body mass index	26.1 ± 4.7	25.6 ± 4.2	0.38
Diabetes mellitus (%)	21.2	28.9	0.25
Cervical JOA score	12.5 (6–17)	11.9 (6–17)	0.22
OP-index	7.1 ± 0.5	10.5 ± 0.6	<0.001 ***
Prevalence of symptoms (%)			
Neck pain	59.8	58.9	0.94
Back pain	25.8	30.8	0.52
Low back pain	54.5	52.3	0.81
JOA-CMEQ score			
Cervical spine function	68.5 ± 28.2	62.5 ± 28.8	0.10
Upper extremity function	81.8 ± 20.6	78.0 ± 22.7	0.19
Lower extremity function	69.0 ± 29.5	62.3 ± 31.9	0.10
Bladder function	76.5 ± 19.8	72.0 ± 24.4	0.11
Quality of life	49.3 ± 20.0	50.7 ± 20.0	0.60
JOA-BPEQ score			
Lumbar spine function	72.3 ± 28.2	62.5 ± 35.0	0.02 *
Social dysfunction	57.7 ± 28.6	54.6 ± 30.4	0.47
Mentality	49.3 ± 19.5	49.0 ± 20.6	0.90
Locomotive function	67.1 ± 33.0	60.6 ± 37.7	0.19
Body pain	71.8 ± 32.7	69.6 ± 34.9	0.63
VAS score			
Neck pain	39.1 ± 30.2	38.4 ± 32.5	0.83
Upper extremity numbness	47.5 ± 32.8	42.1 ± 33.8	0.20
Chest constriction	11.1 ± 22.2	9.2 ± 21.3	0.48
Numbness below the chest	35.3 ± 32.7	39.0 ± 35.6	0.41
Low back pain	25.8 ± 26.6	30.0 ± 31.6	0.29
Lower extremity numbness	29.5 ± 32.7	32.8 ± 35.1	0.47
Lower extremity pain	24.0 ± 30.5	22.0 ± 29.9	0.57

Data are expressed as the mean ± standard deviation or as the percentage. BPEQ, Back Pain Evaluation Questionnaire; CMEQ, Cervical Myelopathy Evaluation Questionnaire; DISH, diffuse idiopathic skeletal hyperostosis; JOA, Japanese Orthopaedic Association; OP-index, ossification of the posterior longitudinal ligament index; OPLL, ossification of the posterior longitudinal ligament; VAS, visual analog scale.; * Significant at *p* < 0.05; *** significant at *p* < 0.001.

**Table 2:** Demographics of patients with cervical OPLL according to DISH grade.

	Grade 1 (*n* = 23)	Grade 2 (*n* = 65)	Grade 3 (*n* = 19)	*p*-Value
Age (years)	65.4 ± 12.7	66.9 ± 12.3	72.8 ± 9.9	<0.001 ***
Male (%)	78.3	75.4	78.9	0.74
Body mass index	25.7 ± 5.0	25.9 ± 3.7	24.7 ± 4.9	0.55
Diabetes mellitus (%)	30.4	32.3	15.8	0.41
Cervical JOA score	12.4 (7.5–17)	11.8 (−2, 17)	11.9 (6–16)	0.36
OP-index	8.7 ± 1.1	10.4 ± 0.8	12.6 ± 1.0	<0.001 ***

Data are expressed as the mean ± standard deviation or as the percentage, DISH, diffuse idiopathic skeletal hyperostosis; JOA, Japanese Orthopaedic Association; OP-index, ossification of the posterior longitudinal ligament index; OPLL, ossification of the posterior longitudinal ligament; *** significant at *p* < 0.001.

**Table 3:** Prevalence of symptoms in patients with cervical OPLL according to DISH grade.

	Grade 1 (*n* = 23)	Grade 2 (*n* = 65)	Grade 3 (*n* = 19)	*p*-Value
Prevalence of symptoms (%)				
Neck pain	78.3	56.9	36.8	<0.05 *
Back pain	30.4	32.3	26.3	0.65
Low back pain	60.9	53.8	36.8	0.14

Data are expressed as the mean ± standard deviation or as the percentage, DISH, diffuse idiopathic skeletal hyperostosis; OPLL, ossification of the posterior longitudinal ligament; * Significant at *p* < 0.05.

## Data Availability

The data generated and analyzed in this study are available from the corresponding author upon reasonable request.
